# Exploring Positive and Negative Affect as Key Indicators of Life Satisfaction among Centenarians: Does Cognitive Performance Matter?

**DOI:** 10.4061/2011/953031

**Published:** 2011-08-18

**Authors:** Alex J. Bishop, Peter Martin, Leonard Poon, Mary Ann Johnson

**Affiliations:** ^1^Department of Human Development and Family Science, Oklahoma State University, 233 HES, Stillwater, OK 74074, USA; ^2^Gerontology Program, Iowa State University, 1085 Elm Hall, Ames, IA 50011, USA; ^3^Institute of Gerontology, University of Georgia, 255 E. Hancock Avenue, Athens, GA 30602, USA; ^4^Department of Foods and Nutrition, University of Georgia, 143 Barrow Hall, Athens, GA 30602, USA

## Abstract

The aim of this investigation was to determine how cognitive performance was associated with positive and negative affect and life satisfaction over time. This study involved a secondary longitudinal analysis of cross-section data collected at Phase I (1988–1992) and during an 18-month longitudinal followup at Phase II (1992–1998) of the Georgia Centenarian Study. Participants included *N* = 137 centenarians at Time 1 and *N* = 68 survivors at Time 2. Significant stability in cognitive impairment existed at Time 1 and Time 2 for positive (*β* = .55, *P* < .01) and negative affect (*β* = .54, *P* < .01) models. Negative affect at Time 1 was associated with lower life satisfaction at Time 1 (*β* = −.42, *P* < .01 ). In addition, cognitive impairment at Time 2 was associated with decreased positive emotionality at Time 2 (*β* = −.39, *P* > .01). Furthermore, greater positive affect at Time 2 was associated with greater satisfaction with life at Time 2 (*β* = .35, *P* < .01). It appears that positive emotionality contemporaneously influences the association between cognitive impairment and life satisfaction among centenarians. Implications relative to improving life satisfaction among centenarians are discussed.

## 1. Introduction

Late adulthood represents a developmental period of contentment in life. Persons surviving to advanced old age are reported to be happier and more satisfied with life than any other age group [1]. This may be due to the fact that old-old adults are effective at diminishing negative affective conditions but optimizing emotionally meaningful life situations [2]. Long-lived persons who have achieved a greater sense of happiness and satisfaction in living have done so precisely for the reason that they have developed the necessary emotional resources to counteract most negative age-associated stressors that may occur near the end of life [3]. For example, old-old adults who have maintained a sense of security in life possessed emotionally gratifying social relations and preserved a favorable view of health positively appraise life [2, 3]. Yet cognitive functioning remains an essential determinant of subjective well-being among persons living 100 years and longer [4–7]. Investigators have acknowledged that normative age-graded cognitive decline suppresses key markers of biopsychosocial well-being including individual autonomy, social involvement, and functional capacity among very old adults [8–11]. This suggests that poor cognitive performance may alter how exceptionally long-lived persons appraise life. However, the extent to which cognitive functioning and emotional affect work in tandem and are associated with life satisfaction among centenarians is not clearly understood. A better understanding of the association between cognition and life satisfaction has implications relative to improving perceived quality of life in extreme longevity. 

### 1.1. Theoretical Considerations

According to Butler [12], reaching advanced old age can create a heightened awareness of impending mortality. Butler [12] theorized that this influences a “naturally occurring and universal process characterized by the progressive return to consciousness of past experience…to maintain one's sense of personal invulnerability” (p. 66). In other words, life review is an individual act and solitary process. Butler [12] hypothesized that old-old adults have a greater amount of unoccupied time to disengage from normative social involvement in order to gauge their well-being. In effect, contemplation was believed to bring about a sense of failure or fulfillment in life. 

However, a disengagement perspective assumes a reduction in ego energy which contributes to subjective well-being in old age. This assumption fails to address the developmental context in which older adults pursue happiness. Erikson [13, 14] recognized late adulthood as the eighth stage of development. During this developmental period, older adults seek some fundamental degree of acceptance for the life, regardless of how fulfilling or challenging it may have been [12]. Erikson [13, 14] argued that sociodevelopmental experiences in later life (e.g., retirement, death of a spouse, family members or close friends, failing health) contribute to a sense of mortality which can precipitate the manifestation of acceptance. In turn, a key developmental task in late adulthood involves active resolution of the life career [14]. Erikson [14] posited that congruence between past failings and accomplishments relative to present life circumstances or conditions of well-being elicits a sense of egointegrity or life satisfaction in later life [14]. Thus, happiness is derived through the positive resolution of the past.

Progression of modern theoretical thinking on subjective well-being over the last three decades has focused on resolving how hedonic qualities contribute to happiness and satisfaction with life [15]. Hedonic well-being is conceptually defined as characteristics that make life satisfying and pleasurable versus dissatisfying and unpleasant [15, 16]. Life satisfaction, the presence of positive affect, and the absence of negative affect are traditionally identified as the core components of hedonism [15, 17] In particular, Diener et al. [18] theorized that positive affect and negative affect influence differential outcomes in life satisfaction relative to individual adaptation to everyday stressors. Based on this theoretical premise, a key assumption is that affective emotions represent adaptive resources that neutralize emotionality and establish a “set-point” of life satisfaction [19]. 

 Recent theoretical and empirical interpretation on subjective well-being has led some investigators to conclude that life satisfaction is derived from cognitive-affective processes that work in tandem to make life pleasurable. According to Socioemotional Selectivity Theory [20, SST], individuals are guided by social (e.g., the feeling of being needed and wanted by others) and emotional (e.g., the need to expand ones horizons or seek meaningful life experiences) goals. As persons reach very late life, they are believed to perceive the remaining years of life as limited. 

This motivates them to engage more frequently in reprioritizing present-oriented life goals. In turn, greater activation and use of cognitive resources occur over a shorter period of time. It is assumed that many old-old adults are disadvantaged by a reduced cognitive reserve necessary for managing everyday challenges that require greater personal attention or decision-making skills (e.g., management cognitive impairment). SST assumes that emotional behavior regulation assumes primacy in the lives of very old adults. Therefore, SST posits that long-lived persons seek to avoid negative emotions and maximize positive affect to alleviate aversive physical or mental health-related symptoms that may compromise satisfaction with life [21, 22]. Such behaviors are believed to free remaining cognitive resources that could potentially be used for more emotionally meaningful and satisfying life activities (e.g., interactions with loved ones). Thus, persons derive satisfaction from life not simply because they may encounter age-associated impairment and seek to age disability or disease-free [3, 23]. Rather, they effectively manage limited psychosocial resources for the purpose of regulating negative emotions in order to enhance feelings of happiness and sense of meaning near the end of life [3, 7].

### 1.2. Happiness and Life Satisfaction

Emotionally meaningful life experiences have a lasting influence on subjective well-being [19]. Past suffering and lifetime achievement contribute to current life satisfaction among old-old survivors [24, 25]. Although many old-old adults view their past as the least satisfying period of life, “right now” is perceived with greater feelings of emotional contentment [25]. This may reflect a cosmic dimension of gerotranscendence in which exceptionally old adults live and rejoice in the moment without dwelling upon past failures [26]. Thus, it is reasonable to assume that old-old adults may be more inclined to savor and recount meaningful life experiences to optimize feelings of happiness. 

Life satisfaction has commonly been recognized as an affective-cognitive construct [3, 4, 11, 17, 18]. The developmental past has been reported as a critical “anchor period” and key mediating indicator of positive emotionality [24, 27]. Cognitive appraisal of the past is an adaptive strategy by which old-old adults diminish feelings of unhappiness [27]. Investigators have acknowledged that old-old adults adapt to challenges and threats to personal well-being by maintaining optimistic and meaningful perceptions of ongoing life experiences [24, 27]. In effect, such perspectives have been reported to be emotionally beneficial relative to emotional happiness in life [27]. Therefore, it can be argued that feelings of discontentment compromise subjective well-being, whereas feelings of emotional contentment may bolster satisfaction with life.

### 1.3. Cognitive Functioning

It is important to note that older adults have a reported preference for recalling positive autobiographical information [28, 29]. Demtsen and Rubin [29] reported that aging adults are twice as likely to report feeling happy than they are to admit to feeling unhappy about life. The tendency to remember the good over the bad is associated with cognitive performance [30, 31]. Better cognitive functioning in advanced old age is associated with greater emotional feelings of meaning and happiness which in turn improves ratings of life satisfaction [3, 11, 29]. In other words, emotional affect can be considered an adaptive resource that regulates the association between cognition and satisfaction in life [28]. Perhaps, cognition is a key determinant of life satisfaction in exceptionally old age relative to the presence of affective emotions.

 Centenarians typically demonstrate great dispersion and variation relative to cognitive performance [3]. Investigators have previously estimated that approximately 20–25% of centenarians can generally be considered as cognitively intact [32, 33]. Another 42–100% are reported to have a considerable degree of dementia [33]. With advancing old age, human beings are more vulnerable to terminal decline or “sudden drop in performance” relative to cognition [34, page 306]. The terminal decline hypothesis specifies that intraindividual change in cognitive performance moves from a preterminal phase of gradual or normative age-associated to a more pronounced and accelerated age-graded decline [34]. Terminal decline in cognitive functioning has been cited as most prevalent among persons who have lived beyond the limits of normal life expectancy [35]. Survival into exceptional old age restricts time left to live. Proximity to death is associated with notable losses in intellectual and cognitive functioning as well as notable changes in subjective well-being believed to remain stable into very old age [36–40]. Variation in associated facets of subjective well-being persists not only over long periods of time but can occur within much shorter time frames [41]. In effect, some investigators have called for greater examination of how associated factors of terminal decline (e.g., cognitive impairment) contribute to perceived subjective well-being as well as emotional-based outcomes of life satisfaction [36–38, 40]. 

The purpose of this investigation was to determine how cognitive performance is associated with positive and negative affect and satisfaction in life over time. In particular, we sought to answer a key question: how is cognitive performance associated with affect and life satisfaction in exceptional old age? We established two key hypotheses. First, we hypothesized that greater cognitive impairment would diminish feelings of positive affect but increase negative affect over time. Second, we hypothesized that greater cognitive impairment would erode how satisfied centenarians feel about life over time.

## 2. Method

This investigation involved a secondary analysis of longitudinal data originating from the Phase I and Phase II Georgia Centenarian Study [32, GCS]. Phase I of the GCS was conducted from 1988 to 1992 and involved a baseline cross-sectional investigation at Time 1. Phase II of the GCS was completed from 1992 to1998 and involved an 18 month longitudinal followup of original and surviving Phase 1 participants. Both phases of the study were reviewed and approved by the university Institutional Review Board (IRB).

### 2.1. Participants Sampling and Procedures

Participants were required to be cognitively intact, community-dwelling, and residing within private residences in the state of Georgia. All participants were cognitively screened and interviewed by a trained interviewer of GCS. Phase I participants consisted of a convenience sample of *N* = 137 during initial assessment at Time 1. Phase 2 participants included a total of *N* = 68 longitudinal survivors who were reassessed and interviewed again 20 months later at Time 2.

All participants were required to be cognitively intact in order to participate. Screening for cognitive status was completed to address two primary considerations: (a) protection of cognitively frail centenarians with advanced cognitive impairment who may not be able to complete a semistructured interview; (b) protection of centenarians from unnecessary stress, fatigue, agitation, or confusion arising during the completion of research involving a *semistructured* interview that would otherwise be beyond their normal daily routine. The Mini-Mental State Examination [42] was the primary instrument used to initially screen all Phase I participants for cognitive status. We considered cognitively intact participants appropriate for study participation to be those with a score of 23 or higher on the Mini-Mental State Examination, as mentioned by Folstein et al. [42]. The Global Deterioration scale [43] served as a secondary cognitive screening instrument. The GDS was used in the event centenarian participants presented with sensory impairments (e.g., vision, hearing) that limited accurate assessment or the ability to fully complete the MMSE. The GDS is a seven-item interviewer rating of subjective memory complaint, orientation, and functional ability. A cut-off score of 2 or less, as mentioned by Reisberg et al. [43], was considered indicative of severe cognitive impairment. All participants were reassessed using the MMSE and the GDS during Phase II 18-month followup.

Demographic characteristics reported by sample participants at Time 1 and longitudinal survivors at Time 2 have been summarized in Table 1. These data were considered for purposes of clarifying whether comparative demographic differences or similarities existed between Time 1 respondents and Time 2 survivors. Special attention was given toward reported marital status, education, and income. Sample variation in these variables has been reported to have underlying and potentially confounding influences in the interpretation of how reported emotionality and life satisfaction may be reported [41]. The majority of participants reported being widowed, having achieved less than a high school education, and earning a low yearly income. Of the follow-up sample, approximately 85.5% were widowed, 64.6% had attained some high school or less, and 72.1% earned an annual income of less than $7,000. To assess differences between respondents in the first and second wave of the study, we computed cross-tabulations. The results suggested that centenarian participants at both waves did not differ relative to marital status *χ* ^2^ (*N* = 134) = 3.97, *P* = .26, education *χ* ^2^ (*N* = 134) = 5.10, *P* = .64, or annual income *χ* ^2^ (*N* = 105) = 5.10,  *P* = .88. Therefore, centenarians within this sample were homogeneous relative to marital status, education, and income at Time 1 and Time 2.

### 2.2. Analytical Design

The main objective of this study was to examine how cognitive impairment was associated with positive and negative affect and life satisfaction across two time points. To achieve this goal, we conducted a secondary longitudinal analysis of Phase 1 (Time 1) and Phase 2 (Time 2) GCS data using SPSS 17.0. Path analytic techniques were used to assess stability coefficients, examine path coefficients between variables, and assess cross-lag effects among variables measured across the two time points [44]. In addition, path analytic models were used to identify any key mediating associations [44]. For this study, we assessed variables reflecting cognitive performance, positive and negative affect, and life satisfaction. 

### 2.3. Measures

#### 2.3.1. Cognitive Performance

Cognitive performance was assessed using the Short-Portable Mini-Mental Status Questionnaire [45, SPMSQ]. The SPMSQ was designed as a brief ten-item test of organic brain functioning. The questionnaire is used to determine orientation to time (e.g., what is the date today?) and place (e.g., what is the name of this place?), recall of information (e.g., when were you born?), and numeric ability (e.g., subtract 3 from 20 and keep subtracting backward). A score of 0–2 typically represents normal cognitive functioning, whereas a score of 3-4 errors indicates mild cognitive, a score of 5–7 errors indicates moderate cognitive impairment, and a score of 8 or more errors is suggestive of severe cognitive deficit. The SPMSQ is also adjusted by education. Pfeiffer [45] noted that participants who have a grade school education are allowed one or more errors. However, one less error is allowed if the person received an education beyond high school. For purposes of this study, we used a summary score indicative to total number of errors made on the SPSMQ. A high cumulative number of errors made represented greater cognitive impairment, whereas a low cumulative number of errors represented less cognitive impairment. Cronbach's alpha of this scale was  .58 at Time 1 and  .73 at Time 2.

#### 2.3.2. Positive/Negative Affect

The Bradburn Affect Balance Scale [46, BABS] served as the primary measure of positive and negative affect. This scale includes two sets of questions, each consisting of five items. One set of questions is used to measure positive affect (e.g., feeling pleased about having accomplished something; feeling on top of the world), whereas the other set of questions is used to evaluate negative affect (e.g., feeling depressed or very unhappy; feeling vaguely uneasy). Participants were asked to indicate how they had recently felt on a four-point Likert scale (1 = not at all; 4 = often). Positive and negative affective items were coded and summed into a summary of positive and negative affect. A high score on positive affective items indicated high positive emotionality, whereas a low score represented low positive emotionality. Similarly, negative affective items were coded so that a high score reflected high negative emotionality and a low score represented low negative emotionality. Test-retest reliability indicative of Cronbach alpha for positive affect items at Time 1 and Time 2 was *α* = .51 and *α* = .66, respectively, whereas test-retest reliability of negative affect items at Time 1 and Time 2 was *α* = .68 and *α* = .60.

#### 2.3.3. Life Satisfaction

The Life Satisfaction Index-A [47, LSI-A] was used as the primary evaluation of life satisfaction. The LSI-A is a 20-item scale used to assess five characteristics of life satisfaction including zest for living (e.g., the things I do are as interesting to me as they ever were); resolution and fortitude (e.g., I have gotten more breaks in life than most people I know); self-concept (e.g., I feel my age but it does not bother me); congruence (e.g., as I look back on my life, I am fairly well satisfied); mood tone (e.g., I am as happy as when I was younger). Participants were asked to indicate whether they 1 = disagree, 0 = are uncertain, or 3 = agree with each statement. Items were summed to form a cumulative score of life satisfaction. A high score indicated high life satisfaction. A low score reflected low feelings of happiness. Alpha reliability across subscale items was  .61 at Time 1 and  .61 at Time 2.

## 3. Results

Means and standard deviations on all study variables were examined to determine whether Time 1 respondent and Time 2 survivors significantly differed relative to responses on all key study variables. These data have been summarized in Table 2. A significant difference in cognitive performance emerged among survivors, *t*(41) = − 4.25, *P* < .01. In other words, average number of errors made on the SPMSQ [32] by respondents at T1 (*M* = 2.07, SD = 1.70) was significantly different from the number of errors surviving respondents made at T2 (*M* = 3.30, SD = 2.35). In particular, surviving respondents made a greater average number of errors at T2. This suggests that longitudinal survivors had diminished cognitive abilities during the 18-month follow-up assessment. In addition, there were no significant mean differences in negative affect, *t*(41) = .57, *P* = .57. This suggests that the average negative affect score of respondents at T1 (*M* = 8.37, SD = 3.24) did not significantly differ from the mean negative affect score of survivors at T2 (*M* = 8.87, SD = 3.27). However, there was a significant difference between respondents at Time 1 and those retested at T2 on average scores of positive affect, *t*(41) = 2.87, *P* < .05. This suggests that average positive affect scores among survivors at T2 were greater than at T1. In particular, average positive affect scores between respondents at T1 (*M* = 12.42, SD = 3.10) and T2 survivors (*M* = 11.26, SD = 3.60) were different. Finally, there was no significant difference evident in the average mean scores of respondent life satisfaction at T1 versus T2, *t*(41) = − .29, *P* = .77. 

**Figure 1:**
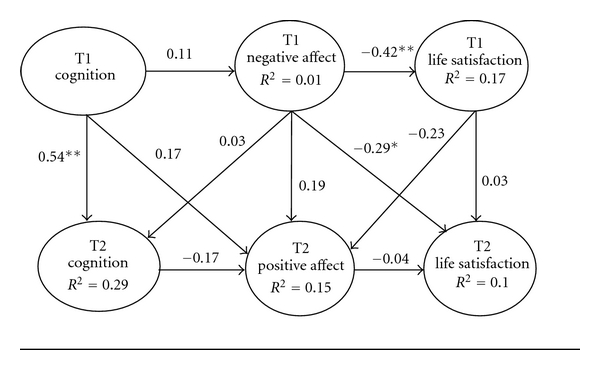
Negative affect model of life satisfaction among centenarians at Time 1 (*N* = 136) to Time 2 (*N* = 68). Note. *P < .05, **P < .01.

A model of negative affect was then constructed and path relationships were assessed (Figure 1). We first examined stability and cross-lag coefficients. Significant stability existed between cognitive impairment at Time 1 and Time 2 (*β* = .54, *P* < .01). Thus, it appears that cognitive impairment remains relative stable over time. Significant stability did not emerge across the negative affect or life satisfaction at Time 1 and Time 2. Slight evidence of a significant cross-lag effect did emerge between negative affect at Time 1 and Time 2 (*β* = − .29, *P* = .05). This suggests that greater negative emotions at T1 decrease life satisfaction at T2. 

**Figure 2:**
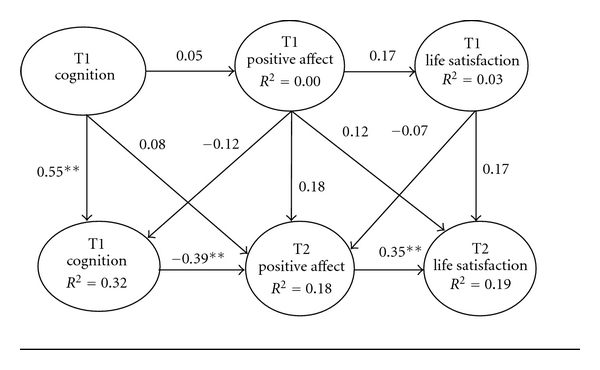
Positive affect model of life satisfaction among centenarians at Time 1 (*N* = 136) to Time 2 (*N* = 68). Note. **P < .01.

Similarly, we assessed path relationships within a panel-design model of positive affect (Figure 2). Significant stability existed between cognitive impairment at Time 1 and Time 2 (*β* = .55, *P* < .01). Thus, it appears that cognitive impairment remains relative stable over time. Significant stability did not emerge across positive affect or life satisfaction at Time 1 and Time 2. Furthermore, there was no evidence of significant cross-lag effects within the positive affect model.

Next, we evaluated path associations between study variables at Time 1 in both models. A significant positive association emerged relative to negative affect and life satisfaction during Time 1 (*β* = − .42, *P* < .01, Figure 1). In particular, centenarians possessing greater negative emotions during the initial assessment also felt less satisfied with life. Seventeen percent of variance in life satisfaction at Time 1 was explained by negative affect at Time 1. Relative to positive affect at Time 1, no significant association emerged with life satisfaction at Time 1. Evidence of a nonsignificant association at Time 1 may represent an anomaly of the study design that was used. Exclusion of cognitively impaired participants may have restricted the range of cognitive ability scores among study participants at Time 1. In turn, this may have led to lower associations between cognitive impairment and positive or negative affect. Finally, only 3% of the variance in life satisfaction at Time 1 was explained by positive affect at Time 1. 

We then examined path coefficients at Time 2 for both models. No significant path associations were evident between cognitive impairment at Time 2 and negative affect at Time 2, or negative affect at Time 2 and life satisfaction at Time 2. However, greater cognitive impairment at Time 2 was associated with reduced positive emotionality at Time 2 (*β* = − .39, *P* < .01). In other words, poor cognitive functioning diminishes the extent to which centenarians experience positive emotions. Nonetheless, greater positive emotions at Time 2 was associated with greater life satisfaction at Time 2 (*β* = .35, *P* < .01). It is important to note that approximately 32% of the variance in cognitive impairment at Time 2 was explained by cognition and positive affect at Time 1. Only 18% of the variance in positive affect at Time 2 was explained by cognitive impairment at Time 1 and 2 as well as positive affect at Time 1. Furthermore, 19% of the variance in life satisfaction at Time 2 was explained by positive affect at Time 1 and Time 2 as well as life satisfaction at Time 1. 

Finally, we consider potential mediating influences. According to Kenny et al. [48], mediation exits when two conditions are met: (1) the path between a predictor and a mediating variable represents a significant increase, while the path from the mediating variable maintains a significant decrease; (2) the path between a predictor variable and a proximal mediating variable represents a significant decrease, while the path from the mediating variable results in a significant increase. 

Based on results from the panel model, two mediating relationships emerged within the positive affect model. First, cognitive impairment at Time 2 represented a key mediating influence between cognitive impairment at Time 1 and positive affect at Time 2. In effect, cognitive impairment at Time 1 continues to erode at Time 2, which in turn diminishes the extent to which centenarians experience positive emotions. Thus, cognition at Time 1 continued to have a negative indirect effect on positive affect in the presence of continued decline in cognitive performance at Time 2 (.55 × −.39 = −.21). Second, positive affect at Time 2 represented a key mediating variable in the association between cognitive impairment and life satisfaction at Time 2. In other words, cognitive impairment at Time 2 continues to compromise current life satisfaction only to the extent centenarians experience decreased positive affect ( −.39 × .35 = − .14). Thus, greater cognitive impairment appears to reduce feelings of emotional happiness which in turn further diminishes life satisfaction among centenarians.

## 4. Discussion

This study provided key evidence to answer the original study question as well as support the original hypotheses. In particular, it appears that cognitive impairment does compromise the extent to which centenarians feel satisfied with life. Two themes emerged based on this finding. First, positive emotions among long-lived persons are associated with continuous decline in cognitive abilities. Second, cognitive impairment has a negative contemporaneous influence on life satisfaction. However, the extent of this association seems to be dependent on whether centenarians are emotionally happy. 

In previous work, we have reported that centenarians who feel emotional contentment generally feel satisfied with life [5, 49]. Yet results from this current investigation suggest that this association may depend on cognitive impairment. In particular, it appears that cognitive impairment over time is detrimental to positive emotionality. As lower cognitive changes persist, current positive affective emotions among centenarians decrease. 

However, the extent to which cognitive impairment is associated with life satisfaction among centenarians may be due to the degree they feel emotionally happy. In other words, the greater positive emotions appear to diminish the deleterious influence of cognitive impairment on life satisfaction among persons living 100 years and beyond. Such evidence may reflect the developmental patterns in life satisfaction in exceptional old age. Erikson [50] referred to extreme late life as the “ninth stage.” In particular, Erikson [50] theorized that living beyond the normative limits of expected human longevity and experiencing on-going physical or mental deterioration promotes a “shift in meta perspective, from a materialistic and rational vision to a more cosmic and transcendent one, normally followed by an increase in life satisfaction” [50, p. 123]. Investigators have acknowledged the association between positive emotionality and life satisfaction as an adaptive behavior unique to long-lived persons [51]. As centenarians continue to live, they encounter advanced and terminal declines in physical and mental functioning. Such challenges may tax their ability to use psychosocial resources for purposes of adaptation and improved disposition toward life. From the framework of socioemotional selectivity theory [20, 21], it is plausible to argue that centenarians are experts relative to knowing how to selectively dissipate negative emotions and use positive emotional energies to optimize sense of fulfillment in life despite investigators have noted that the interplay between cognition and emotionality is vital source of coping and resilience in old-old age [3]. Thus, the achievement of emotional happiness and life satisfaction among centenarians can be argued as a normative developmental outcome of surviving diminished cognitive abilities. 

Gerotranscendence provides an additional explanation of findings. In devising the theory of gerotranscendence, Tornstam [26] posited that the past provides a sense of coherence and gives meaning to the present. This may represent a developmentally normative task of exceptional longevity. Old-old age brings an anticipation of impending loss in cognitive, physical, or functional capacity [35]. Nonetheless, old-old adults remain resilient by adapting new strategies in response to age-associated decline [27]. Contemplation of the accomplishments and traumas of life allows persons in very old age to return with an emancipated comprehension or new appreciation toward self and others [26]. In effect, the joy of appraising and resolving one's life career may serve to lift exceptionally old persons out of despair and regret and into a state of emotional contentment  [26, 52, 53]. Lucas [19] has hypothesized that adaptation to alternating life events across the life evokes stability or a “set-point” in levels of happiness. Old-old persons may experience life-altering events which compromise emotional happiness, yet a positive sense of adaptation to change allows them to eventually gain a heightened sense of satisfaction with life. This might help explain the continued impact of cognitive impairment on positive emotionality among centenarians over time. Furthermore, it may provide a plausible explanation for a contemporaneous and indirect association between cognitive impairment and life satisfaction in the presence of positive emotions. Further investigation of the impairment versus emotional well-being dynamic in old-old age is warranted. 

Subjective well-being represents an affective-cognitive association [17, 41, 53]. Results from this study appear to support this hypothesis relative to positive affect. Pleasant emotions evolve to the degree that persons possess the cognitive resources to create current satisfying memories in the present as well as to recall meaningful experiences of the past [24, 41]. Some investigators have reported that life satisfaction entails autobiographical recall of essential and meaningful life events [54, 55]. In some cases, high degree of reported life satisfaction in old age represents a deactivated negative affective state [56, 57]. This has been acknowledged to further improve cognitive functioning as well as retrieval of positive emotional experiences across the life-span [57, 58]. The occurrence of positive emotions improves adaptive capacity and allows persons in advanced old age to regain locus of control over unhappy situations or compromising life experiences [59, 60]. Suh et al. [61] proposed that most individuals rely directly on cognitive functioning and affective states to frame judgment of life satisfaction. It can be argued that this may further provide support for the contemporaneous association between positive affect and life satisfaction.

However, poor cognitive performance does reduce positive emotions. Rabbitt et al. [11] reported that decline in cognitive capacity contributes to heightened feelings of emotional negativity and unhappiness. The trajectory and rate of cognitive decline and memory deficits becomes more pronounced as persons live beyond the expected limits of human life expectancy [6, 10, 30]. This is exacerbated by accumulative everyday hassles (e.g., degree of autonomy in activities of daily living) and personal irritations (e.g., health problems, social isolation) which often require greater use and performance of executive abilities in old-old age [10, 31]. 

It is important to note that many centenarians possess a limited or finite cognitive reserve to properly counteract age-associated impairment [7, 32]. Impairment in old-old age typically represents noticeable decline in pathological process (e.g., organic brain functioning) which contribute to a simultaneous and terminal drop in overall life satisfaction [36–38, 40]. Yet as persons reach very late life, age-associated impairment becomes secondary, and they perceive their life script more favorably [29, 61]. Investigators have noted that old and very old adults tend to report emotional feelings of contentment twice as often as they endorse feelings of emotional dissatisfaction [29]. Therefore, it is plausible to argue that pleasant emotions remain relatively intact despite increasing cognitive impairment. Perhaps, it is the persistence of positive affective conditions in the face of age-associated impairment that matters most for life satisfaction among long-lived persons.

Nonetheless, our findings do provide support of the detrimental influence poor cognitive functioning has on life satisfaction [11]. In particular, continued cognitive impairment erodes the extent to which centenarians derive favorable impressions of life. Cognitive impairment also appears to have a more contemporaneous association with emotional happiness. In other words, the deleterious influence of cognitive impairment on positive emotions appears to be most salient among those who continue to survive in exceptional old age. Centenarians who have experienced recent cognitive problems feel dissatisfied with life to the extent that they feel happy. Results from this study indicate that impaired cognitive functioning in extreme late life negatively influences a key indicator of life satisfaction, namely, positive affect. This may call into question the potential benefits of life review or reminiscence therapies often used to elicit feeling of emotional happiness and life satisfaction among centenarians [62, 63]. However, this requires further investigation beyond the scope and findings of this study. 

Findings from this study provide insight into the subjective well-being of centenarians. Nonetheless, several limitations should be noted. For instance, convenience sampling was used in selecting a participant sample of community-dwelling centenarians residing in the Southern United States. The final sample used for this investigation also represented cognitively intact centenarians. As previously mentioned, researchers have reported an estimated 42–100% of centenarian study samples with moderate to severe degree of dementia [33]. The incorporation of a randomized and population-based sample may have resulted in improved generalizablilty of results. However, caution should still be used in interpreting results across other centenarian populations which may be cognitive impaired, originate from care facilities, or reside in other regions of the United States or world. 

Another limitation involved interval of assessment. Although we had two time points of assessments for longitudinal analysis, the selection of different intervals between measurements would have enhanced the study. In particular, this may have led to significant time lag effects which would have improved understanding of how long or after what time cognitive impairment, congruence, or happiness exhibit influences. Additional time points would have also allowed for use of more sophisticated methods and use of growth curve modeling. The assessment of life satisfaction across multiple time points has been reported to advance understanding of the developmental change and temporal mechanisms of subjective well-being [41]. 

Finally, the measure of life satisfaction used in this study could be improved in future research. We used only one quantitative measure of life satisfaction, the LSI-A [47]. This may have resulted in considerable overlap in constructs representing past satisfaction with life and current happiness. Furthermore, test-retest alpha reliabilities of the LSI-A were low to marginal. Items from the LSI-A [47] may not provide an appropriate assessment of life satisfaction among persons living exceptionally long lives. Life may be conceptualized differently among centenarians than other age groups [62, 63]. Furthermore, life satisfaction in extreme old age may be a more complex and dynamic phenomenon which requires integration of advance quantitative assessments, qualitative evaluations, or greater focus on cross-cultural comparison for improved scientific understanding. For example, the incorporation of a structured life review or a cross-cultural comparison may have presented alternative findings pertaining to longitudinal outcomes of emotional happiness and life satisfaction among exceptionally old adults. Perhaps, such knowledge would improve scientific understanding of whether life satisfaction among centenarians is universally constructed or culturally unique. In effect, qualitative insight could have resulted in more sophisticated interpretation of subjective well-being. 

Despite limitations, this study established key insights into how cognitive impairment and affect represent key indicators of life satisfaction. The results also raise potential questions pertaining how to effectively enhance feelings of happiness and life satisfaction among cognitively impaired persons in exceptional old age. Researchers should continue to increase their understanding of the underlying mechanisms of cognition associated with life satisfaction in advanced old age. Particularly, investigators should seek to further explore and model conceptual and theoretical longitudinal models of subjective well-being to examine cognitive change and adaptation in the development of happiness and satisfaction with life.

## Figures and Tables

**Table 1:** Frequencies of demographic characteristics among centenarian respondents at Time 1 (*N* = 136) and Time 2 survivors (*N* = 68).

Variable	T1 respondents		T2 survivors	
	*N*	%	*N*	%
Marital status				
Single	9	6.7%	5	8.1%
Married	6	4.5%	3	4.8%
Widowed	116	86.6%	53	85.5%
Divorced	3	2.2%	1	1.6%
Total	134	100.0%	62	100.0%
Education				
0–4 years	19	14.1%	11	16.1%
4–8 years	39	28.8%	23	33.8%
Some high school	16	11.9%	10	14.7%
High school	13	9.6%	4	5.9%
Business/trade	8	5.9%	4	5.9%
Some college	19	14.1%	7	10.3%
College	12	8.9%	5	7.4%
Graduate school	9	6.7%	4	5.9%
Total	135	100.0%	68	100.0%
Income				
$1,000–1,999	2	1.9%	2	3.7%
$2,000–2,999	4	3.8%	3	5.6%
$3,000–3,999	11	10.5%	9	16.6%
$4,000–4999	20	19.0%	10	18.5%
$5,000–5,999	24	22.9%	11	20.3%
$6,000–6,999	10	9.5%	4	7.4%
$7,000–9,999	15	14.3%	7	13.0%
$10,000–14,999	6	5.7%	3	5.6%
$15,000–19,999	2	1.9%	—	—
$20,000–29,999	6	5.7%	3	5.6%
$40,000 and over	5	4.8%	2	3.7%
Total	105	100.0%	54	100.0%

**Table 2:** Descriptive statistics of T1 respondent sample (*N* = 136) and Time 2 survivor sample (*N* = 68).

Variable	T1 respondents		T2 survivors	
	*M*	SD	*M*	SD
Cognitive impairment	2.07	1.70	3.30	2.35**
Positive affect	12.42	3.10	11.26	3.60*
Negative affect	8.37	3.24	8.87	3.27
Life satisfaction	12.37	3.09	12.41	3.11

Note. *P < .05. **P < .01
